# Deficiency of T-Cell Intracellular Antigen 1 in Murine Embryonic Fibroblasts Is Associated with Changes in Mitochondrial Morphology and Respiration

**DOI:** 10.3390/ijms222312775

**Published:** 2021-11-26

**Authors:** Isabel Carrascoso, Beatriz Ramos Velasco, José M. Izquierdo

**Affiliations:** Centro de Biología Molecular ‘Severo Ochoa’, Consejo Superior de Investigaciones Científicas-Universidad Autónoma de Madrid (CSIC-UAM), C/Nicolás Cabrera 1, Cantoblanco, 28049 Madrid, Spain; i.martinez.carrascoso@gmail.com (I.C.); bramos@cbm.csic.es (B.R.V.)

**Keywords:** TIA1, mitochondria, mitochondrial dynamics, mitochondrial respiration, murine embryonic fibroblast

## Abstract

T-cell intracellular antigen 1 (TIA1) is a multifunctional RNA-binding protein involved in regulating gene expression and splicing during development and in response to environmental stress, to maintain cell homeostasis and promote survival. Herein, we used TIA1-deficient murine embryonic fibroblasts (MEFs) to study their role in mitochondria homeostasis. We found that the loss of TIA1 was associated with changes in mitochondrial morphology, promoting the appearance of elongated mitochondria with heterogeneous cristae density and size. The proteomic patterns of TIA1-deficient MEFs were consistent with expression changes in molecular components related to mitochondrial dynamics/organization and respiration. Bioenergetics analysis illustrated that TIA1 deficiency enhances mitochondrial respiration. Overall, our findings shed light on the role of TIA1 in mitochondrial dynamics and highlight a point of crosstalk between potential pro-survival and pro-senescence pathways.

## 1. Introduction

The RNA-binding protein (RBP) T-cell intracellular antigen 1 (TIA1) is involved in many aspects of RNA metabolism and governs the flow of gene expression [1,2]. The TIA1 gene encodes two major mRNA/protein isoforms (known as TIA1a and TIA1b), which are generated by alternative splicing (inclusion and skipping, respectively) of exon 5, which is highly conserved between mice and humans [3], and both isoforms are expressed in a cell- and tissue-specific manner [4,5]. The protein structure of TIA1 contains three RNA recognition motifs (RRMs) that bind to mRNA and a glutamine- and asparagine-rich C-terminal domain with intrinsically disordered regions [1,2,3,4].

TIA1 is a master regulator of the crosstalk between nuclear and cytoplasmic compartments in eukaryotic cells [1,2]. For example, it modulates both constitutive and alternative splicing [6,7,8], aids in the transport and subcellular localization of mRNAs [9,10,11] and is responsible for the stability and translation of mRNAs [12,13,14,15,16] by direct interaction or competition with other proteins and RNAs [16,17,18,19,20]. TIA1 recognizes U-, UC-, and/or AU-rich sequences located on the non-consensus 5′ and/or 3′ splice sites of introns on pre-mRNAs, on the 5′ and 3′ untranslated regions (UTRs) of mRNAs and along with the sequences of non-coding RNAs [16,17,18,19,20]. It is thought that TIA1 interacts potentially with 5–10% of the coding and non-coding genes of proteins that are synthesized from the human genome [18,19,20].

Changes in the expression and/or subcellular localization of TIA1 have been associated with important pathophysiological consequences in human biology and disease, including embryogenesis [14,21,22], inflammation [14,23,24], tumorigenesis [25,26,27], neuronal homeostasis [28,29], tauopathies [30], myopathies [31,32,33,34,35], cell stress [10,11,12] and viral infections [2,36]. The participation of TIA1 in these complex programs points to its direct involvement in the regulation of myriad cellular pathways among other apoptosis [1,25], autophagy/mitophagy [21,26,37], immune system [13,24,38], membrane dynamics [39], axonal regeneration [40], activity and localization of cellular translational machinery [17,41], cell cycle [2,19,24,29], proteostasis [42,43], dynamics of stress granules during environmental challenges (oxidative, heat, osmotic, etc.) [9,10,11,43,44] and mitochondrial dynamics [21,45,46].

Targeted ablation of TIA1 in mice leads to high embryonic lethality [14], but the penetrance varies from zero in TIA1-knockout mice on the C57Bl/c background [47] to ~50% on the BALB/c background [14]. Adult TIA1-KO mice show a mild-arthritis phenotype [14] and recapitulate several key features of chronic post-traumatic stress disorder in humans. This phenotype is observed predominantly in female mice [29], and TIA1 haploinsufficiency exacerbates neuroinflammation in tauopathy [31,48].

The role of TIA1 in key cellular events, such as inflammation and the stress response is well recognized, but less is known about its involvement in early development-related cellular programs. In this work, we approach the characterization of the mitochondrial respiratory phenotype associated with the TIA1 knocked-out murine embryonic fibroblasts (MEFs). Here, we show that TIA1 expression facilitates mitochondrial dynamics and respiration in MEFs.

## 2. Results

**Figure 1:**
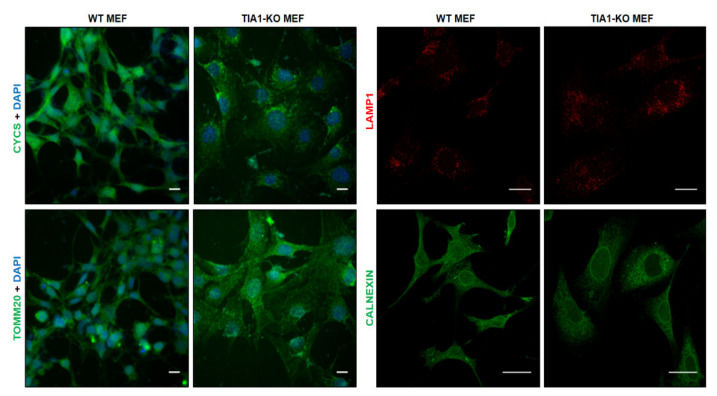
Mitochondrial morphology in wild-type and TIA1-knock out mouse embryonic fibroblasts. Mitochondrial spatial morphology in wild-type (WT) and TIA1 knock out (TIA1-KO) mouse embryonic fibroblasts (MEFs) by confocal fluorescence microscopy. Immunofluorescence images from WT and TIA1-KO MEFs. The antibodies used were against cytochrome c (CYCS, green), translocase of outer mitochondrial membrane 20 (TOMM20, green), lysosomal associated membrane protein 1 (LAMP1, red), and calnexin (CANX, green) proteins. In panels indicated as CYCS and TOMM20, the nuclei were stained with DAPI. Bars represent 10 μm (CYCS and TOMM20 images), and 20 μm (LAMP1 and CANX images).

Comparative analysis of the mitochondrial phenotype in wild-type (WT) and TIA1-KO MEFs revealed apparent morphological changes in the organized mitochondrial network, as determined by immunostaining against the mitochondrial components cytochrome c (CYCS) and translocase of the outer mitochondrial membrane 20 (TOMM20) (Figure 1, upper panel). Immunostaining of lysosomal-associated membrane protein 1 (LAMP-1, middle panel), a glycoprotein used as a lysosomal marker, showed an increase in the number of lysosomes in TIA1-KO MEFs (Figure 1, middle panel), which agrees with previous findings [21]. By contrast, the relative expression of calnexin (CANX), a calcium-binding, endoplasmic reticulum-associated protein, that interacts transiently with newly synthesized N-linked glycoproteins, (facilitating protein folding and assembly) was unaffected by the deficiency in TIA1 (Figure 1, lower panel). 

**Figure 2:**
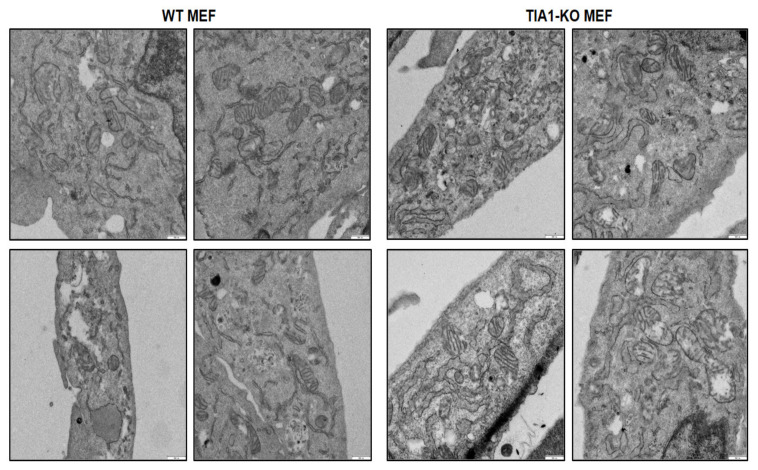
Mitochondrial architecture in wild-type and TIA1-knock out mouse embryonic fibroblasts. Details of mitochondrial morphology and cristae architecture in in wild-type (WT) and TIA1-knock out (TIA1-KO) mouse embryonic fibroblasts (MEFs) were visualized by transmission electron microscopy. White bars represent 500 nm.

An analysis of mitochondria using transmission electron microscopy (TEM) revealed an altered mitochondrial architecture in TIA1-KO cells, including heterogeneous mitochondrial populations with some elongated mitochondria and abnormal cristae densities and sizes (Figure 2). These results suggest that TIA1 could shape mitochondrial spatial dynamics and are compatible with enhanced mitochondrial fusion and cristae remodeling.

**Figure 3:**
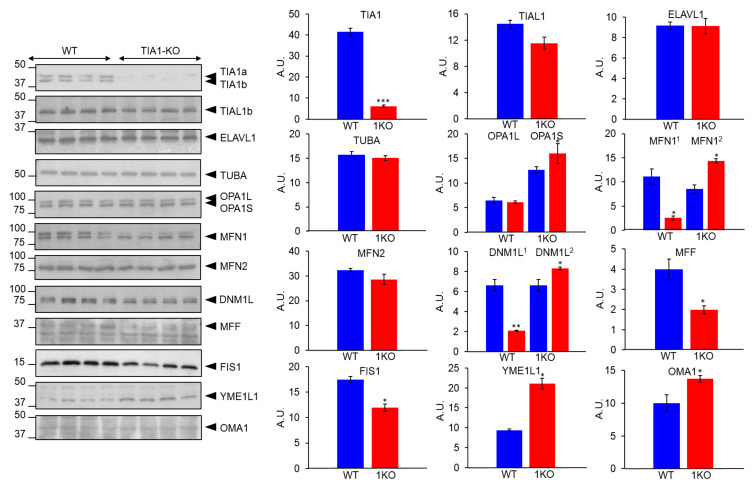
Proteomic analysis of mitochondrial proteins associated with mitochondrial morphology and dynamics in wild-type and TIA1-knock out mouse embryonic fibroblasts. Protein extracts from wild-type (WT) and TIA1-knock out (TIA1-KO) mouse embryonic fibroblasts (MEFs) were analyzed by western blotting using specific antibodies against the indicated proteins. Molecular weight markers for proteins (kDa) and the identities of proteins are indicated: T-cell intracellular antigen 1 (TIA1), TIA1-related protein (TIAL1), ELAV-like RNA binding protein 1 (ELAVL1), alpha subunit of tubulin (TUBA), optic atrophy protein 1 (OPA1), mitofusin 1 (MFN1), mitofusin 2 (MFN2), dynamin 1 like (DNM1L), mitochondrial fission factor (MFF), mitochondrial fission 1 protein (FIS1), YME1 like 1 ATPase (YME1L1) and OMA1 zinc metallopeptidase (OMA1). The bar chart represents the relative abundance (arbitrary units) of the analyzed proteins in homogenates from WT (blue bars) and TIA1-KO (1KO, red bars) MEFs. A.U. means arbitrary units. Values are mean + SEM (*n* = 4; * *p* < 0.05; ** *p* < 0.01; *** *p* < 0.001).

To better understand the role of TIA1 in the genetic control of mitochondrial fusion and/or fission, we assessed the relative abundance of several proteins involved in this dynamic process using cell extracts of WT and TIA1-KO MEFs. Comparative western blotting analysis revealed changing patterns of mitochondrial components involved in fusion dynamics, such as the ratio of the (long) L and (short) S variants of optic atrophy protein 1 (OPA1), and the differential electrophoretic mobility of mitofusin 1 (MFN1) (Figure 3) [49,50,51]. Likewise, we observed differential patterns of dynamin 1 like (DNM1L) protein (upper and lower bands) and a lower expression of mitochondrial fission factor (MFF) and mitochondrial fission 1 (FIS1) proteins in TIA1-KO MEFs; both are well-characterized molecular events associated with the suppression of mitochondrial fission processes [50,51]. In addition, the expression of the metallopeptidases ATP-dependent zinc metalloprotease (YME1L1) and OMA1 zinc metallopeptidase (OMA1) was moderately higher in TIA1-KO MEFs than in WT counterparts (Figure 3). As expected, TIA1 expression was absent in the KO-MEFs, and the expression of TIA1-related protein (TIAL1/TIAR) and Hu antigen R (ELAVL1/HuR) was unchanged. 

**Figure 4:**
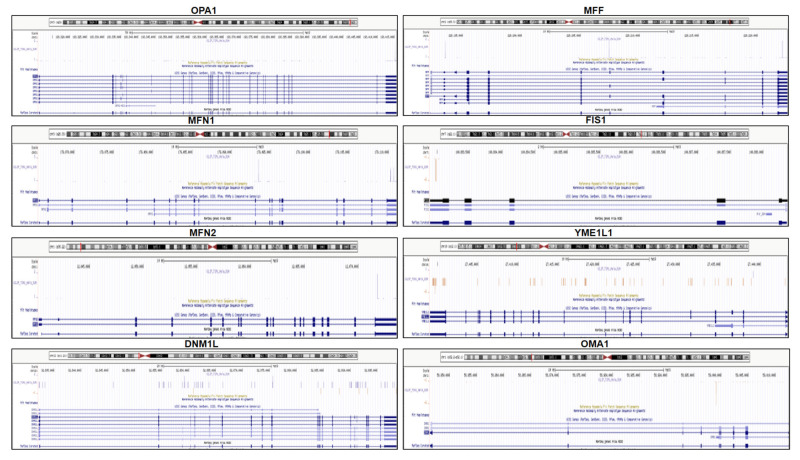
In Vivo cross-linking sites of TIA1-iCLIP at specific nuclear-encoded mitochondrial pre-mRNAs associated with mitochondrial organization/dynamics. The RNA map, corresponding to TIA1 binding on indicated human genes, was adapted using TIA1-iCLIP analysis [18]. The localization of target genes on human chromosomes and the exon and intron positions of the human pre-mRNAs are shown. The bar graph in each panel indicates the number of cDNAs identified in each TIA1 crosslinking site. The following human genes are shown: optic atrophy protein 1 (OPA1), mitofusin 1 (MFN1), mitofusin 2 (MFN2), dynamin 1 like (DNM1L), mitochondrial fission factor (MFF), mitochondrial fission 1 protein (FIS1), YME1 like 1 ATPase (YME1L1) and OMA1 zinc metallopeptidase (OMA1).

The interactions of TIA1 on the transcripts of the aforementioned fusion/fission-related genes were investigated using individual-nucleotide resolution UV cross-linking and immunoprecipitation (iCLIP), which revealed specific sites of in vivo TIA1 binding along their pre-mRNAs (Figure 4). 

**Figure 5:**
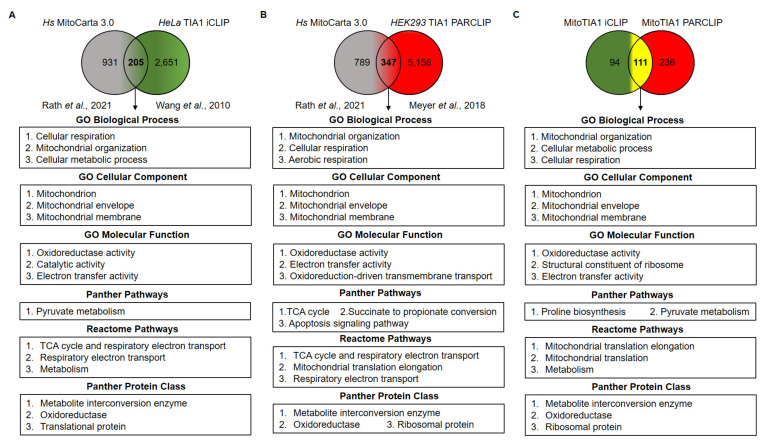
Gene Ontology analysis of nuclear-encoded mitochondrial genes associated with human TIA1-iCLIP and PARCLIP database. (**A**) Venn diagrams displaying number of nuclear-encoded mitochondrial genes (*Homo sapiens*) MitoCarta 3.0) [48] and HeLa TIA1 iCLIP analysis [18]. (**B**) Venn diagrams displaying number of nuclear-encoded mitochondrial genes (*Homo sapiens*) MitoCarta 3.0) [49] and HEK293 TIA1 PARCLIP analysis [19]. The shared mitochondrial genes/pre-mRNAs between both TIA1 iCLIP (**A**) and PARCLIP (**B**) analysis were identified and classified using the PANTHER database. (**C**) Venn diagrams showing shared nuclear-encoded mitochondrial genes between both TIA1-iCLIP and TIA1-PARCLIP analysis and GO categories using PANTHER tool.

These findings likely explain the observations on mitochondrial dynamics/morphology and architecture associated with TIA1 deficiency and confirm and extend previous data [21,45,46]. As TIA1 deficiency has functional consequences on mitochondrial morphology, we expanded our study to investigate whether other potential nuclear-encoded mitochondrial genes were targeted by TIA1. To this end and given the experimental gap in the mouse database on the specific transcriptome of murine TIA1-binding sites, we performed a comparative study using the latest version of MitoCarta [49] containing all human mitochondrial genes and in vivo binding sites for TIA1 substantiated from experimental TIA1-iCLIP [18] (Figure 5A) and photoactivatable ribonucleoside-enhanced crosslinking and immunoprecipitation (PARCLIP) [19] biochemical analysis (Figure 5B). The results indicated that of 1136 human nuclear-encoded mitochondrial genes (Figure 5A,B) versus the selective transcriptome of 2856 and 5505 human pre-mRNAs with TIA1 binding sites by iCLIP and PAR-CLIP analysis, respectively, 205 and 347 pre-mRNAs (18–30.5% of human nuclear-encoded mitochondrial genes) were potential targets of TIA1 (Figure 5A,B, and Figures S1 and S2). In total, 111 genes were shared between the iCLIP and PARCLIP analysis (Figure 5C and Figure S2).

To examine the shared human nuclear-encoded mitochondrial genes and identify relevant associated biological processes, selected genes were analyzed by PANTHER classification (http://pantherdb.org, accessed on 1 November 2021). Pathways enriched among the gene ontology (GO) categories identified from the shared human and mouse mitochondrial genes included biological processes (cellular respiration, mitochondrial organization and cellular metabolic processes), cellular components (mitochondrial envelope and membrane), molecular functions (oxidoreductase, catalytic and electron transfer activities), PANTHER-specific and reactome pathways (pyruvate metabolism, tricarboxylic cycle and respiratory electron transport) as well as involved protein classes (metabolite interconversion enzymes, oxidoreductases and mitochondrial translation-related proteins) (Figure 5A,B). Furthermore, when the 111 shared nuclear-encoded mitochondrial genes between the TIA1-iCLIP and PARCLIP methods (Figure 5A,B) were clustered using GO analysis, the results indicated that the biological processes and categories are matched those above filters (Figure 5A–C, and Figures S1 and S2). 

**Figure 6:**
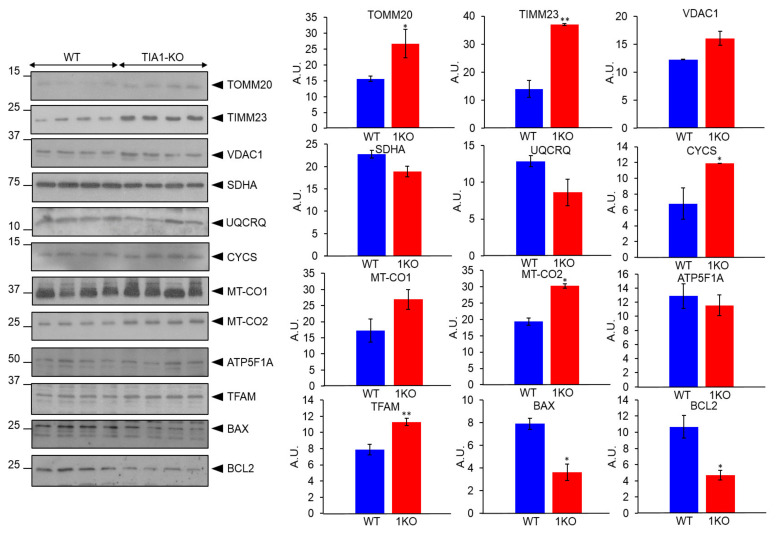
Proteomic analysis of nuclear-encoded mitochondrial proteins associated with mitochondrial envelope, membrane and respiration in wild-type and TIA1-knock out mouse embryonic fibroblasts. Protein extracts from wild-type (WT) and TIA1-knock out (TIA1-KO) mouse embryonic fibroblasts (MEFs) were analyzed by western blotting using specific antibodies against the indicated proteins: translocase of outer mitochondrial membrane 20 (TOMM20), translocase of inner mitochondrial membrane 23 (TIMM23), voltage dependent anion channel 1 (VDAC1), succinate dehydrogenase complex flavoprotein subunit A (SDHA), ubiquinol-cytochrome c reductase complex III subunit VII (UQCRQ), somatic cytochrome c (CYCS), mitochondrially encoded cytochrome c oxidase I (MT-CO1), mitochondrially encoded cytochrome c oxidase II (MT-CO2), ATP synthase F1 subunit alpha (ATP5F1A), mitochondrial transcription factor A (TFAM), apoptosis regulator BCL2 associated X (BAX), and BCL2 apoptosis regulator. Molecular weight markers for proteins (kDa) and the identities of proteins, are indicated. The bar chart represents the relative abundance of the analyzed proteins in cytosol from WT (blue bars) and TIA1-KO (1KO, red bars) MEFs. A.U. means arbitrary units. Values are mean + SEM (*n* = 4; * *p* < 0.05; ** *p* < 0.01).

To gain more evidence on the role of TIA1 in the genetic control of mitochondrial components associated with mitochondrial respiration and function, we quantified the relative abundance of several core proteins in cell extracts of WT and TIA1-KO MEFs. The results revealed significant differences in the abundance of many core proteins, including TOMM20, translocase of inner mitochondrial membrane 23 (TIMM23), cytochrome c (CYCS), mitochondrially encoded cytochrome c oxidase II (MT-CO2) and mitochondrial transcription factor A (TFAM), which were all higher in TIA1-KO MEFs than in WT-MEFs (Figure 6). 

The opposite was observed for the apoptosis regulators B-cell lymphoma 2 (BCL2) and BCL2 associated X (BAX), which were significantly lower (Figure 6). These observations are in accord with the proteomic analysis (Figure 3 and Figure 5) [46,47] and indicate that several mitochondrial functions are associated with metabolic flux, gene expression, respiratory electron transport, import, sorting and dynamics/organization, are modulated by TIA1. The data strongly suggest that other nuclear-encoded mitochondrial genes identified by in silico analysis may be also targeted by TIA1 (Figure S3).

**Figure 7:**
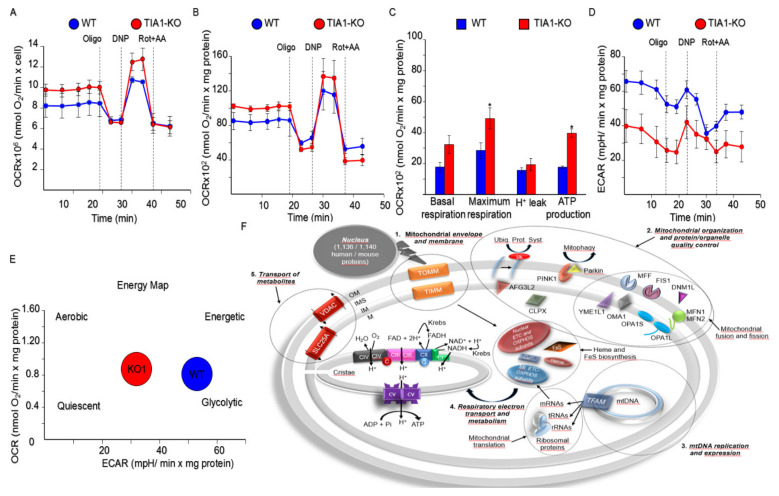
TIA1 deficiency in mouse embryonic fibroblasts enhances mitochondrial respiration. (**A**,**B**) Real-time changes in oxygen consumption rate (OCR) using cells under basal condition (glucose 10 mM) and after sequential injection of oligomycin, 2, 4-dinitrophenol (DNP) and rotenone plus antimycin A. Values were normalized to cell number (**A**) and mg protein (**B**). (**C**) Mitochondrial parameters of non-mitochondrial, basal and maximal respiration, proton (H^+^) leak, ATP production and spare respiratory capacity. Values are mean + SEM (*n* = 3–5; * *p* < 0.05). (**D**) Real-time changes in the extracellular acidification rate (ECAR), an indicator of lactic acid production or glycolysis. (**E**) Determination of the aerobic and glycolytic components of cellular bioenergetics. (**F**) Hallmarks of categories and activities/functions associated with nuclear-encoded mitochondrial proteins potentially targeted by TIA1. The main PANTHER GO categories identified in silico (numbers 1–5) as well as some examples of clustered nuclear-encoded mitochondrial genes and functional categories are included on a schematized mitochondrion. The legends identified as OM, IMS, IM, and M, are for outer membrane, inner mitochondrial space, inner membrane and matrix, respectively. The following acronyms are indicated: AFG3L2 (AFG3 like matrix AAA peptidase subunit 2), CLPX (caseinolytic mitochondrial matrix peptidase chaperone subunit X), DNM1L (dynamin 1 like), FIS1 (mitochondrial fission 1 protein), MFF (mitochondrial fission factor), MFN1 (mitofusin 1), MFN2 (mitofusin 2), OMA1 (zinc metallopeptidase OMA1), OPA1 (optic atrophy protein 1), PINK1 (PTEN induced kinase 1) SLC25A (solute carrier family 25 member), TFAM (mitochondrial transcription factor A), TIMM and TOMM (translocase of inner and outer mitochondrial membrane systems, respectively), VDAC (voltage dependent anion channel), and YME1L1 (YME1 like 1 ATPase).

We hypothesized that the evident differences in the expression of nuclear-encoded mitochondrial components between WT and TIA1-KO MEFs would impact the respiratory phenotype. Specifically, we expected that the observed morphological and gene/protein expression changes in TIA1-KO MEFs would support a more efficient mitochondrial respiration. To test this, we measured the oxygen consumption rate (OCR) in MEFs using the Seahorse Bioscience XF analyzer. The mitochondrial respiratory response was measured before and after stress tests by the sequential addition of oligomycin, 2,4-dinitrophenol, rotenone and antimycin A, to determine ATP-linked, maximal and non-mitochondrial respiration, respectively (Figure 7A,B). 

Basal mitochondrial respiration was significantly higher in TIA1-KO MEFs than in WT MEFs (Figure 7C). Likewise, maximal mitochondrial respiration, ATP production and spare mitochondrial respiration capacity were also significantly higher in TIA1-KO MEFs than in WT MEFs (Figure 7C). By contrast, the extracellular acidification rate (ECAR), an index of glycolysis, was higher in WT MEFs than in TIA1-KO MEFs (Figure 7D), indicating that anaerobic glucose oxidation is lower in TIA1-KO MEFs (Figure 7E). These findings suggest that deficiency of TIA1 in MEFs has a positive effect on mitochondrial electron transport chain function and respiration.

In summary, our functional analysis together with previous observations indicates that TIA1 binding sites are associated with the following mitochondrial hallmarks: (1) mitochondrial envelope and membrane processes (i.e., protein sorting and import), (2) protein/organelle quality control and mitochondrial organization, (3) mitochondrial DNA expression and replication, (4) respiratory electron transport and mitochondrial metabolism, and (5) transport of metabolites (i.e., inorganic ions, protons, amino acids, nucleotides, coenzymes, cofactors, etc.) (Figure 7F).

## 3. Discussion

Our previous results in MEFs deficient for TIA1 revealed compromised cell proliferation concomitant with a delay in cell cycle progression (G2/M phase) and resolution of cell division [21]. We also found that TIA1-KO MEFs had an increase in the number of mitochondrial DNA copies measured as the ratio of mitochondrial/nuclear DNA, which may be the result of a decrease in mitochondrial fission and/or an increase in mitochondrial fusion [21]. Our present study extends this analysis by revealing changes in the proteomic patterns of specific molecular components related to mitochondrial fission and fusion, as well as to the intra-mitochondrial architecture itself. This is illustrated by the expression changes of essential factors involved in mitochondrial fission, such as DNM1L, MFF, and FIS1, as well as in fusion-associated components, such as OPA1 (OPA1S *versus* OPA1L) and MFN1. The expression patterns are consistent with decreased mitochondrial fission or division, favoring mitochondrial fusion. The mitochondrial morphological changes are also consistent with the increase in mitochondrial respiratory and regulatory components, which fits well with the increase in mitochondrial respiratory capacity and accords with the increase in mitochondrial reactive oxygen species (ROS) and in oxidative damage of mitochondrial DNA we previously observed in TIA1-KO MEFs [21]. These events linked to mitochondrial dynamics and functionality might be an adaptive survival response in the form of adaptive autophagy to alleviate cell damage and prevent the development of deleterious phenotypes, ensuring cell viability according to previous findings [16,21,26,45,46]. 

An interesting question emerges based on our observations: could the cellular expression and/or location of TIA1 regulate many nuclear-encoded mitochondrial genes? A simple answer to this might be that these mRNAs or their precursors (pre-mRNAs) could be targeted by TIA1 through one or multiple layers to exert control of their gene expression; thus, this multifunctional regulator can act as an RBP in several subcellular scenarios [9,10,11,12,13,14,15,16,17,18,19,20]. For example, as shown in the present study, the pre-mRNAs analyzed have multiple sequence sites spanning their full length, both exons and introns, and for which we have detected both high and moderate densities of TIA1 binding sites. This suggests a potential post-transcriptional regulation given the density of binding sites on around 197 nuclear-encoded mitochondrial pre-mRNAs derived from TIA1-iCLIP analysis and located with some frequency on the introns and last exons of the pre-mRNAs, and particularly on the sequences located at the 5′/3′ splice sites of introns and 3′ untranslated regions of the pre-mRNAs. In this regard, it is reasonable to imagine the existence of a feedback loop that activates and/or represses the expression of many genes, which could be activated and/or repressed in the absence or presence of TIA1, for example, at the post-transcriptional level of mRNA stability/turnover and/or translational activation/repression, to dampen their expression in order to promote or counteract the cellular and/or mitochondrial phenotypes associated and/or modulated to the expression, subcellular location, and/or functional post-translational modifications of TIA1 and its isoforms during homeostasis and stress conditions. 

Mitochondria morphology is dynamic and is controlled by highly ordered events, which have functional consequences [50,51]. Several genetic and environmental conditions are known to enhance mitochondrial fusion/fission; for example, limited nutrient availability favors a hyperfused mitochondrial network that increases ATP production [52] and protects mitochondria against mitophagy [53], which is dependent on mitochondrial fragmentation. Mitochondrial reorganization is also an integral part of the control of cell cycle progression to ensure the correct distribution between daughter cells [54,55,56]. 

Some cellular protective adaptations, for instance, autophagy, cause mitochondrial hyperfusion to prevent mitochondrial degradation [57]. In another protective context, the p53-dependent transient fusion of mitochondria with lysosomes allows mitochondria to escape mitophagy [58], a process linked to cellular Ca^2+^ [59]. Mitochondria (hyper)fusion also occurs in response to oxidative stress and extracellular acidosis and is often referred to as stress-induced mitochondrial hyperfusion. Again, this serves as a protective measure to increase respiratory efficiency and limit cell death [54,60]. Additionally, the balance between stemness and differentiation is related to mitochondrial dynamics and appears to be also tightly linked to the interplay between mitochondria and Ca^2+^ signaling [61,62,63,64]. 

Aging is a major risk factor for many human diseases, such as Alzheimer’s disease, cancer and cardiovascular diseases [65]. Evidence suggests that aging occurs in a regulated manner and that perturbation of discrete cell signaling pathways (nutrient signaling, mitochondrial function, etc.) can extend lifespan and delay age-related diseases [66]. Regulation of mitochondrial dynamics has emerged as an important regulatory hub during aging. For instance, the RBP Pumlio 2 (PUM2) prevents MFF-mediated mitochondrial dynamics and associated mitophagy during aging [67]. In fact, PUM2 (and its ortholog in *C. elegans* PUF-8) regulates the translation of MFF mRNA. PUM2/PUF-8 is overexpressed during and represses MFF expression, which prevents mitochondrial fission. Inhibition of mitochondrial fission represses the mitophagy response to exert the control of quality and replacement of deleterious mitochondria by promoting mitochondrial dysfunction that affects and reduces longevity. This led to the identification of the RBP PUM2 as a negative regulator of longevity and health span in nematode and mouse models.

Both oncogene- and radiation-induced senescence is associated with increased mitochondrial biogenesis, fusion, and reduced mitophagy [68]. The mechanistic link, if any, is incompletely understood but seems likely to involve increased ROS levels. While further evidence is needed, we speculate that inducing senescence is another way in which mitochondrial dynamics could play an important role in cancer cells, because senescent cells are protected from chemotherapy-induced death yet can contribute to cancer development through the senescence-associated secretory phenotype (SASP) [68]. While some previous reports suggest that mitochondrial hyperfusion is initiated to ameliorate cellular stress, the precise mechanism of mitochondrial hyperfusion and its role in maintaining cellular homeostasis as well as its negative impact on cellular health in disease conditions, however, remains unclear [64]. 

Cellular senescence is linked to coordinated programs of gene expression control at the transcriptional and post-transcriptional levels [69,70]. Our study and previous observations [14,24] suggest that ablation of TIA1 in MEFs can lead to a senescence-like phenotype, involving diminished cell growth, delayed cell-cycle progression, nuclear DNA damage and low global rates of *de novo* protein synthesis [14,24]. We show here that TIA1-KO MEFs remain metabolically active with higher respiratory rates than in equivalent proliferating cells, which is also observed during senescence, and is in agreement with the mitochondrial proteomic profiling and morphology results. A typical feature of senescent cells is the production and secretion of a substantial amount of inflammatory proteins as part of the SASP [64]. This complex secretome contains inflammatory cytokines, interleukins, and chemokines, angiogenic growth factors, and tissue-remodeling metalloproteases and insoluble factors of the extracellular matrix [64]. TIA1 regulates the decay and translation of mRNAs encoding a diverse class of proteins, including inducible proinflammatory cytokines, constitutive survival factors, and angiogenic growth-associated proteins [12,13,14,15,16,17]. There are no RNA binding maps of TIA1 in MEFs, but previous findings and the present study (by using large-scale binding and functional maps of human RBPs in human cells) suggest that TIA1 could regulate mRNA stability and RNA decay through varying regulatory mechanisms that likely involve cell-type-specific co-factors [18,19,20,21,22]. Thus, TIA1 could have a functionally versatile role, acting as a dual agent pro-growth or pro-senescence factor during embryonic development, cellular homeostasis and stress, or tumorigenesis in a cellular context-dependent manner [2,24,25,26,27,28].

TIA1 also plays both general and specific roles as a translational repressor in response to environmental stress agents (heat shock, oxidants, hyperosmolarity, etc.) [2,9,10,11]. Under conditions of stress, cells can form non-membranous cytoplasmic structures termed stress granules (SGs). TIA1 possesses three RNA recognition motifs and a prion-related domain through which it can self-aggregate within SGs, hijack ribonucleoprotein complexes, and suppress translation globally and specifically through the interaction with RNAs containing A and U-rich sequence elements (AREs). Thus, TIA1 can directly mediate the translational silencing and turnover of ARE-containing mRNAs and non-coding RNAs (ncRNAs) and can indirectly function as a molecular sponge to modulate the regulatory activity mediated by microRNAs [9,10,11,15,16,17,18,19,20]. Given the presence of TIA1 and other RBPs that determine the fate of many cellular RNAs in SGs, these foci function as dynamic sites of RNA triage during stress, where molecular decisions are made regarding the composition of RNA ribonucleoprotein complexes and their subsequent engagement with the translation or degradation machinery by modulating the cellular and metabolism fate with pathophysiological consequences on normal and abnormal cell growth [71,72,73].

Several RBPs (up to 14 RBPs reviewed in [74]) could directly and/or indirectly modulate (including through their crosstalk) mitochondrial versatility (phenotype and functionality) in several cellular processes. Our results indicate that a deeper mechanistic interrogation of RBP biology (comparing physiological and pathological levels) and their pharmacological regulation are necessary to fully appreciate the biological and clinical implications of this important metabolic and regulatory network. Indeed, recent literature reveals that TIA1-mediated signaling captures a broad spectrum of survival and stress pathways that likely influence its contrasting antagonistic functions as proto-oncogene and tumor suppressor [24,25,26,27,28]. It is becoming increasingly apparent that TIA1 and its isoforms have multiple intersections with numerous cellular processes, which adds further complexity, interest, and broader therapeutic potential to the double life of multifunctional TIA1 in health and disease [2]. 

## 4. Material and Methods

### 4.1. Cell Cultures

WT and TIA1-KO MEFs were generated and maintained as described [14,21].

### 4.2. Immunofluorescence and Electron Transmission Microscopy Analysis

MEFs were processed for immunofluorescence and TEM analysis as described [21]. For immunofluorescence microscopy analysis we used specific antibodies against CYCS (sc-13156 (1/100), Santa Cruz Biotechnology); TOMM20 (sc-17764 (1/100), Santa Cruz Biotechnology, Dallas, TX, USA); LAMP-1 (AB_528127 (1/50), DSHB, Iowa City, IA, USA); and CANX (SPC-108B (1/200), StressMarq, Victoria, BC, Canada).

### 4.3. Western Blotting Analysis

Protein samples were separated by 10% SDS-PAGE and transferred to a nylon membrane at 4 °C and 50 V for 2 h. The membrane was then blocked with 5% powdered milk in phosphate-buffer saline solution (pH 7.5) containing 0.1% Tween-20 (Merck, Darmstadt, Germany) (PBS/T). The membrane was then probed with specific antibodies against the indicated proteins. Membranes were incubated with primary antibodies in PBS/T containing 3% BSA (Sigma) overnight at 4 °C, washed, and then incubated with appropriate HRP-conjugated secondary antibodies at room temperature for 1 h. The ECL reagent (GE Healthcare, Chicago, IL, USA) for HRP was used as a developer. The antibodies used were the following: ATP5F1A (ab14748 (1/2000), Abcam, Cambridge, UK); BAX (sc-493 (1/1000), Santa Cruz Biotechnology, Dallas, TX, USA); BCL2 (sc-492 (1/1000), Santa Cruz Biotechnology, Dallas, TX, USA); CYCS (sc-13156 (1/1000), Santa Cruz Biotechnology, Dallas, TX, USA); DNM1L (33318 (1/1000), Signalway Antibody, College Park, MD, USA); ELAVL1 (sc-5261 (1/4000), Santa Cruz Biotechnology, Dallas, TX, USA); FIS1 (33067 (1/500), Signalway Antibody, College Park, MD, USA); MFF (orb101576 (1/1000), Biorbyt, Cambridge, UK); MFN1 (sc-166644 (1/1000), Santa Cruz Biotechnology, Dallas, TX, USA); MFN2 (sc-100560 (1/1000), Santa Cruz Biotechnology, Dallas, TX, USA); MT-CO1 (GR3338268-2 (1/500), Thermo Fisher, Waltham, MA, USA); MT-CO2 (ab110258 (1/2000), Abcam, Cambridge, UK); OMA1 (sc-168844, (1/1000), Santa Cruz Biotechnology, Dallas, TX, USA); OPA1 (sc-393296 (1/1000), Santa Cruz Biotechnology, Dallas, TX, USA); SDHA (AB14715 (1/1000), Abcam, Cambridge, UK); TFAM (HPA040648 (1/1000), Prointech, Manchester, UK); TIA1 (sc-1751 (1/3000), Santa Cruz Biotechnology, Dallas, TX, USA); TIAL1 (sc-1749 (1/3000), Santa Cruz Biotechnology, Dallas, TX, USA); TIMM23 (ab230253 (1/2000), Abcam, Cambridge, UK); TOMM20 (sc-17764 (1/1000), Santa Cruz Biotechnology, Dallas, TX, USA); TUBA (T5168 (1/5000), Merck, Darmstadt, Germany); UQCRQ (14975-1-AP (1/2000), Proteintech, Manchester, UK); VDAC1 (ab15895 (1/2000), Abcam, Cambridge, UK); and YME1L1 (sc-139302 (1/500), Santa Cruz Biotechnology, Dallas, TX, USA).

### 4.4. Seahorse Analysis

Mitochondrial activity assays and determination of mitochondrial oxygen consumption rates were carried as described [46]. Briefly, cellular OCR was determined on the XF24 Extracellular Flux Analyzer platform (Seahorse Bioscience, North Billerica, MA, USA). Cells were plated on XF24 microplates at 15,000 cells/well in supplemented medium and incubated at 37 °C and 5% CO_2_ for 24 h. After measuring basal respiration, 6 μM oligomycin was injected to inhibit complex V, and then 0.75 mM 2,4-dinitrophenol was injected to uncouple respiration. Finally, respiratory complex I and III were inhibited by injection of 1 μM rotenone and 1 μM antimycin A, respectively. OCR was determined by subtracting the ‘non-mitochondrial OCR’ after treatment with rotenone + actinomycin A, whereas mitochondrial basal respiration was determined from mitochondrial OCR before administration of oligomycin. Mitochondrial maximal respiration was defined as OCR after administration of 2,4-dinitrophenol. Spare respiration capacity was defined as maximal respiration minus basal respiration. The cells shift to an almost exclusive aerobic phenotype as indicated by a low ECAR and the cells shift to a more glycolytic phenotype with an average OCR equal to 20 pmoles/min and an average ECAR equal to 75 mpH/min.

### 4.5. Functional Analysis of Gene Lists

Venn diagrams were constructed using the Venny 2.1.0—Bioinfo GP tool (http://bioinfogp.cnb.csic.es/tools/venny (accessed on 9 September 2021)) from TIA1-iCLIP [18] and the PARCLIP [19] database, comparing them to updated human nuclear-encoded mitochondrial genes in MitoCarta 3.0 [49]. GO database analysis was performed with the PANTHER classification system (http://pantherdb.org, accessed on 1 November 2021). Statistical overrepresentation test was used to identify GO term enrichments in significantly shared genes.

### 4.6. Statistical Analysis

The data were expressed as mean ± SEM. The student’s *t*-test was applied to determine statistical significance between two groups. *p* values < 0.05 were considered statistically significant.

## Data Availability

Not applicable.

## Supplementary Materials

The following are available online at https://www.mdpi.com/article/10.3390/ijms222312775/s1.
