# Supplementary figures and images for: Deficiency of T-Cell Intracellular Antigen 1 in Murine Embryonic Fibroblasts Is Associated with Changes in Mitochondrial Morphology and Respiration
Source: Int J Mol Sci. 2021 Nov 26;22(23):12775. doi: 10.3390/ijms222312775 (PMC8657690; doi:10.3390/ijms222312775)

## TOMM20

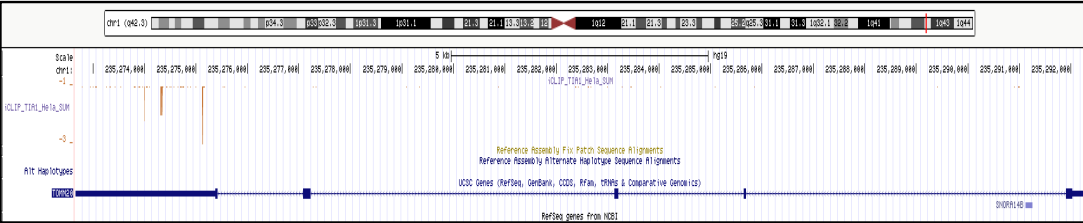

# TIMM23

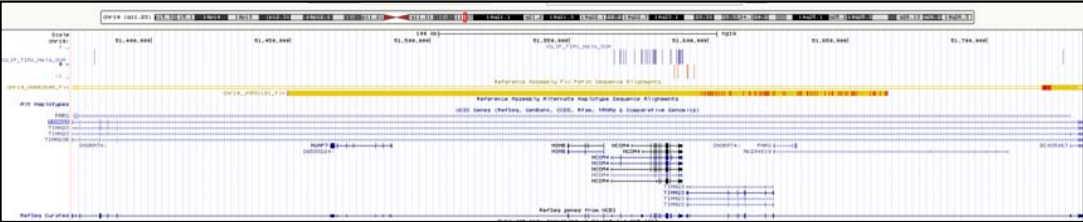

## VDAC1

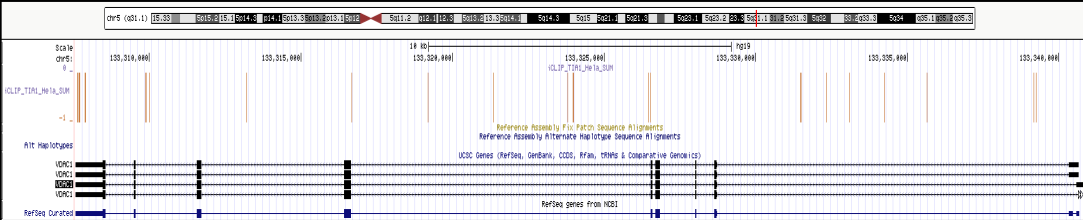

## SDHA

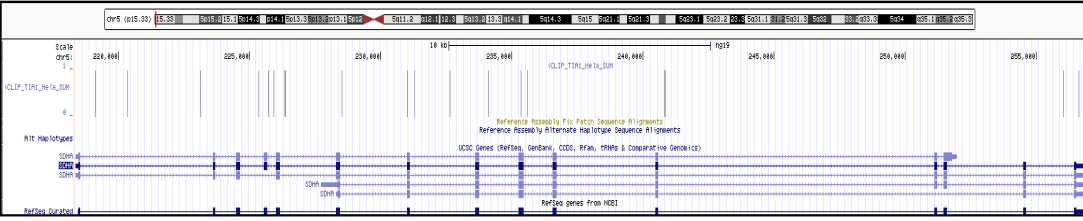

**UQCRQ**

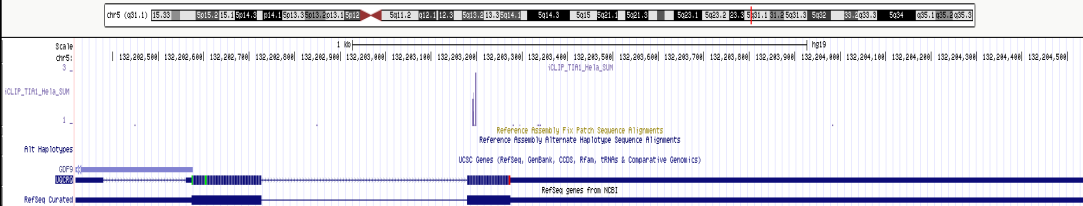

**CYCS**

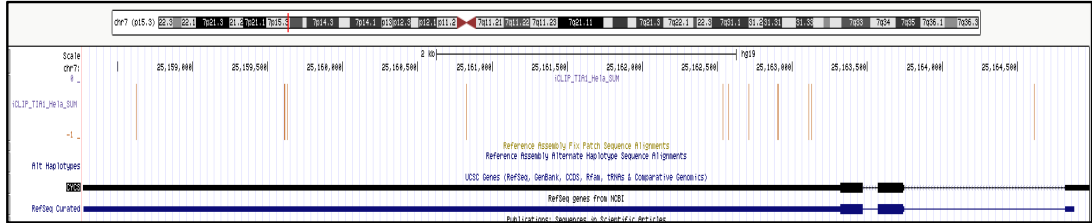

## ATP5F1A

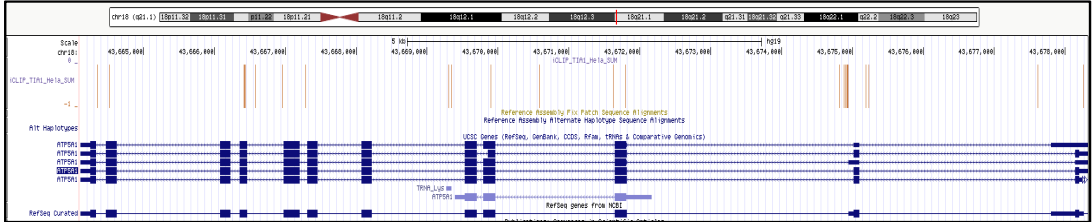

## TFAM

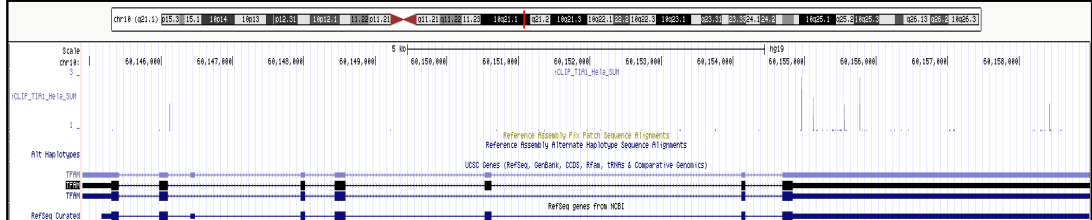

**BAX**

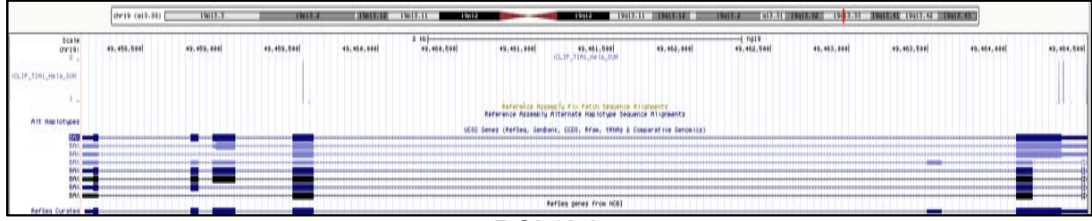

## BCL2L1

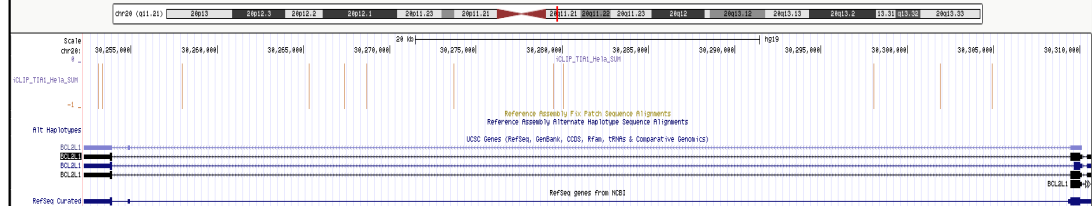

Supplement: Supplementary file 1 [file ijms-22-12775-s001.zip › Figure S3.pdf]

WB – FIGURE 3

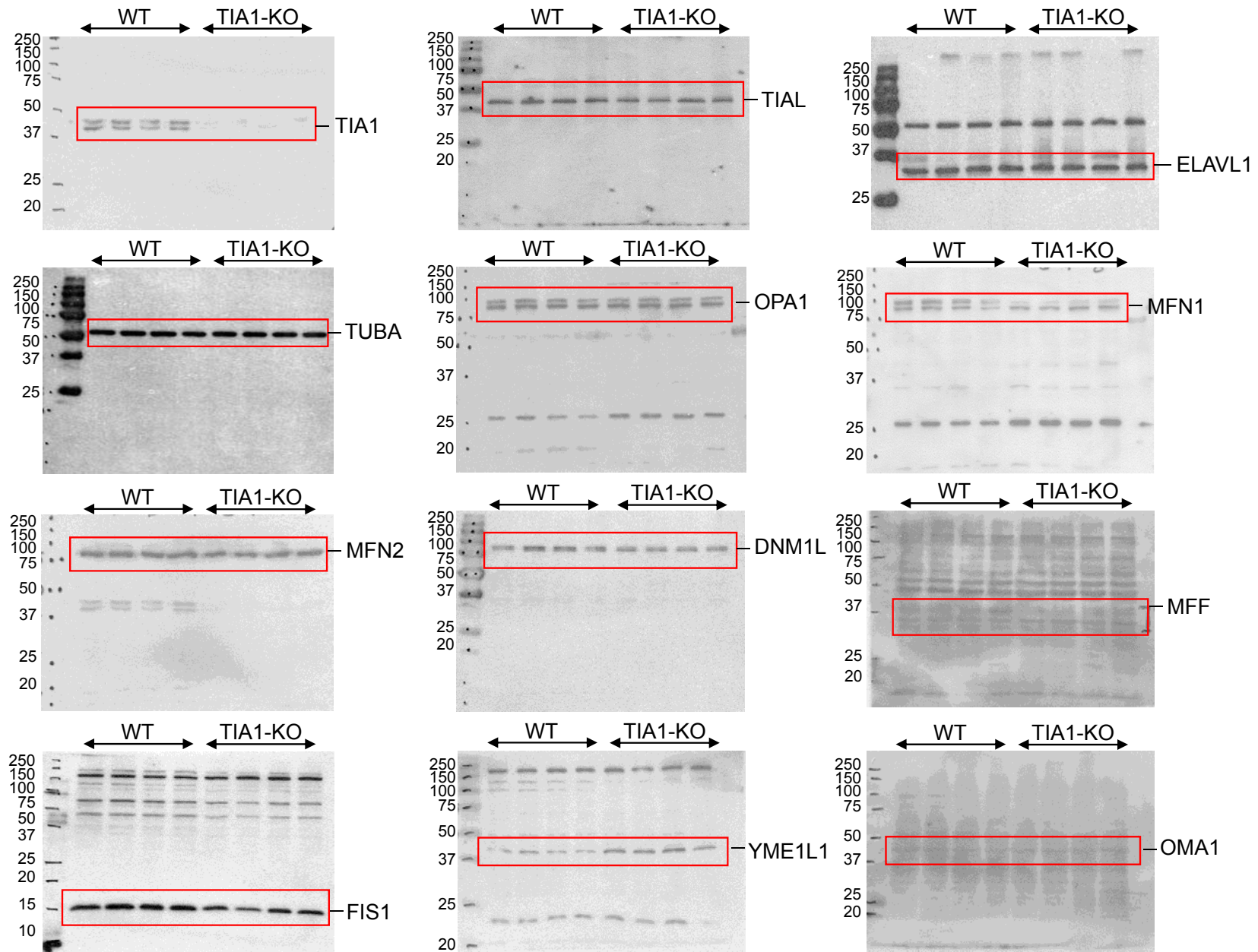

WB – FIGURE 6

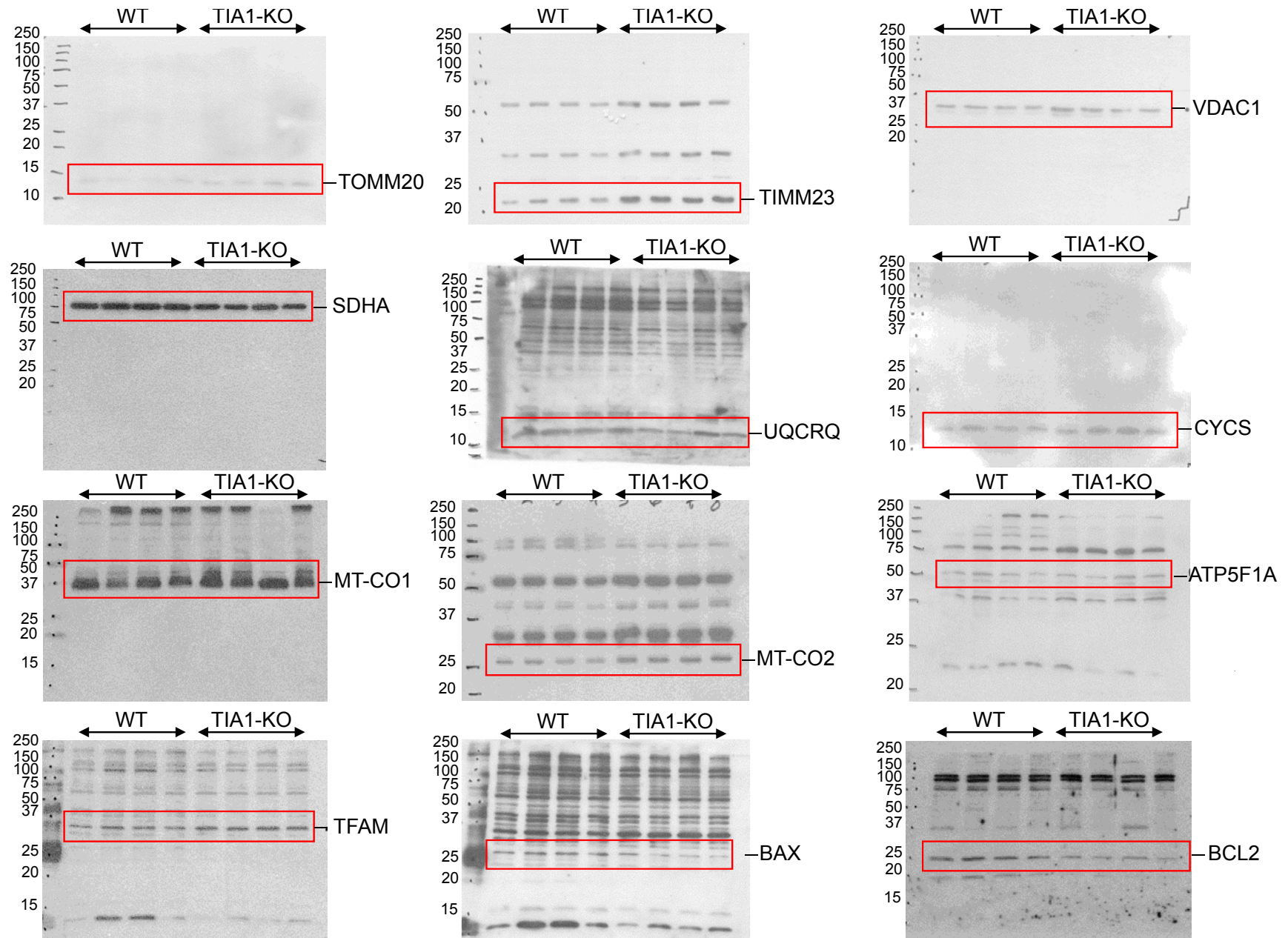

Supplement: Supplementary file 1 [file ijms-22-12775-s001.zip › Original images of WB analysis.pdf]
